# Differential Efficacy of Glycoside Hydrolases to Disperse Biofilms

**DOI:** 10.3389/fcimb.2020.00379

**Published:** 2020-07-24

**Authors:** Whitni K. Redman, Garrett S. Welch, Kendra P. Rumbaugh

**Affiliations:** ^1^Department of Surgery, Texas Tech University Health Sciences Center, Lubbock, TX, United States; ^2^Immunology and Molecular Microbiology, Texas Tech University Health Sciences Center, Lubbock, TX, United States; ^3^TTUHSC Surgery Burn Center of Research Excellence, Texas Tech University Health Sciences Center, Lubbock, TX, United States

**Keywords:** *Pseudomonas aeruginosa*, *Staphylococcus aureus*, biofilms, dispersal, glycoside hydrolase, chronic wounds

## Abstract

Chronic wounds will impact 2% of the United States population at some point in their life. These wounds are often associated with a reoccurring, chronic infection caused by a community of microorganisms encased in an extracellular polymeric substance (EPS), or a biofilm. Biofilm-associated microbes can exhibit tolerance to antibiotics, which has prompted researchers to investigate therapeutics that improve antibiotic efficacy. Glycoside hydrolases (GHs), enzymes that target the polysaccharide linkages within the EPS, are one potential adjunctive therapy. In order to develop GH-based therapeutics, it is imperative that we understand whether the composition of biofilm EPS changes based on the environment and/or presence of other microbes. Here, we utilized α-amylase and cellulase to target the polysaccharides within the EPS of mono- and dual-species *Pseudomonas aeruginosa* and *Staphylococcus aureus* biofilms in three different models that vary in clinical relevancy. We show that biofilms established in an *in vitro* well-plate model are not strongly adhered to the polystyrene surface and do not accurately reflect the GH efficacy seen with biofilms grown *in vivo*. However, dispersal efficacy in an *in vitro* wound microcosm model was more reflective of that seen in a murine wound model. We also saw a striking loss of efficacy for cellulase to disperse *S. aureus* in both mono- and dual species biofilms grown in the wound models, suggesting that EPS constituents may be altered depending on the environment.

## Introduction

Bacteria were previously thought to predominately live in a free-floating or “planktonic” mode of life. However, advancements in microbiology within the last 50 years have led to an understanding of the natural community lifestyle of aggregated microorganisms now referred to as “biofilms.” Biofilms, defined as communities of bacteria encased in a complex matrix of biopolymers, provide shelter for their constituent bacteria. This shelter inherently provides increased tolerance to desiccation, nutrient limitation, antibiotics, and phagocytosis (Flemming and Wingender, 2010; Solano et al., 2014). The proximity of biofilm-associated bacteria can also lead to increased bacterial coordination through quorum sensing and an increased frequency of horizontal gene transfer (Flemming and Wingender, 2010), further increasing the fitness of the biofilm's population. These advantages make biofilm infections difficult for the immune system to clear, even with clinical intervention, and can lead to chronic, recalcitrant infections. Since experts estimate that 80% of all human bacterial infections are biofilm-associated (Romling and Balsalobre, 2012) and biofilm-associated bacteria have demonstrated over a 1,000-fold increase in antimicrobial tolerance (Rogers et al., 2010), many researchers have begun pursuing alternative treatment methods for these infections.

One methodology focuses on targeting the extracellular polymeric substance (EPS). The EPS surrounds the biofilm-associated microbes and is composed of exopolysaccharides, proteins, lipids, and extracellular DNA (eDNA). Because the EPS represents over 90% of the dry mass in most *in vitro* biofilms and provides the structural scaffolding for the three-dimensional architecture (Flemming and Wingender, 2010), targeting the EPS will theoretically reduce the integrity of the biofilm, eliminating the increased tolerance to antimicrobials and host immune responses. Our previous results demonstrated that one class of EPS degrading enzyme, glycoside hydrolases (GHs), could effectively break-up *Pseudomonas aeruginosa* (PA) biofilms and disperse cells, which were then easier to kill with antibiotics (Fleming et al., 2017; Fleming and Rumbaugh, 2018). GHs target the integral linkages in polysaccharides, breaking them into simple sugars.

While GHs show promise for treating biofilm-associated infections, in order to be an effective therapeutic, GHs would need to break up biofilms produced in a wide variety of physiological environments, including *in vivo* microenvironments that have differing nutritional profiles, oxygen levels and pHs, and that may harbor different microbial species. Unfortunately, the majority of our understanding of EPS structures is based on the biofilms formed by a limited number of species grown *in vitro*. We have very limited knowledge about the composition of EPS when multiple species are present or if it changes based on the environment in which biofilms are grown. One reason for this gap in knowledge is that determining the biochemical components of the EPS of biofilms is technically challenging and has only been reported for a limited number of mono-species biofilms grown *in vitro*. Thus, determining the EPS components of a bacterial biofilm growing *in vivo* and differentiating those components from the surrounding host material would be a monumental task.

In order to try to understand if and how the EPS changes in different environments, we sought to determine if the efficacy of GHs differed when PA and *Staphylococcus aureus* (SA) biofilms were grown alone or together in different environments. We used two GHs, α-amylase (from *Bacillus subtilis*), which targets α- 1,4 linkages, and cellulase (from *Aspergillus niger*), which targets β- 1,4 linkages. PA produces 3 main exopolysaccharides. Pel, which possess α- 1,4 linkages, should be targeted by amylase. Alginate and Psl both possess β- 1,4 linkages (Ma et al., 2009; Yang et al., 2011), thus should be targeted by cellulase. The polysaccharide made by SA (poly *N*-acetyl glucosamine (PIA/PNAG) on the other hand does not possess linkages targeted by the GHs chosen, and thus should be resistant to treatment (Arciola et al., 2015). By utilizing GHs that target these specific linkages we sought to determine if polysaccharide production differs depending on the model in which bacteria are grown or between mono and poly-microbial biofilms.

## Materials and Methods

### Bacterial Strains and Growth

*P. aeruginosa* strain PAO1 (Holloway et al., 1979) and *S. aureus* strain SA31 (Watters et al., 2014) have been previously described. PA and SA were grown in baffled 250 mL Erlenmeyer flasks, with shaking at 200 rpm in Luria-Bertani/Lysogeny Broth (LB) broth at 37°C for 16–18 h overnight. 150 μL of the overnight culture was used to inoculate a sub-culture containing 10 mL of LB broth and was grown at 37°C at 200 rpm for 2.5 h. Planktonic cells in the sub-culture were then used to initiate biofilms. Viable cells were quantified by serial dilution and 10 μL spot plating on *Staphylococcus* medium 110 (Difco) and *Pseudomonas* isolation agar (Difco) as colony forming units (CFU).

### Glycoside Hydrolases

Bacterial α-amylase (from *Bacillus subtilis*, 02100447; MP Biomedicals, LLC) and fungal cellulase (from *Aspergillus niger*, 02150583; MP Biomedicals, LLC) were utilized for these experiments. All enzymes were prepared by dissolving lyophilized powder in 1x phosphate buffered saline (PBS) at 37°C for 30 min. 1x PBS was used as a vehicle control.

### *In vitro* Well-Plate Dispersal

To measure percent dispersal *in vitro*, the wells of a 24-well non-tissue culture-treated plate (Falcon) were inoculated with 10^5^ CFU (in 800 μL) subcultured SA, PA, or SA and PA together. For poly-microbial biofilms, 20% of the inoculating solution was adult bovine serum (B9433 Sigma-Aldrich®), to prevent PA from outcompeting SA as previously shown (Smith et al., 2017). Biofilms were grown for 48 h at 37°C with shaking at 80 rpm. Following incubation, the supernatant was removed, and each well was gently rinsed with 1 mL PBS to dislodge any non-adhered cells. Subsequently, wells were treated with 1 mL of the enzyme solutions or a vehicle control for 2 h at 37°C with shaking at 80 rpm. Following treatment, the supernatant was removed and serially diluted in PBS. CFU were enumerated to calculate the “dispersed” fraction. 1 mL of PBS was added to the remaining biofilms, which were broken up by 30 min of sonication, then serially diluted and spot plated for CFU enumeration to determine the “biofilm” fraction. Percent dispersal was calculated by dividing the dispersed CFU by the total CFU (biofilm-associated plus dispersed). Viable cells were quantified by serial dilution and 10 μL spot plating on *Staphylococcus* medium 110 (Difco) and *Pseudomonas* isolation agar (Difco) as colony forming units (CFU).

### Microcosm Model

To measure percent dispersal in an *in vitro* assay that mimics the wound environment, sterile, glass, 1 mL test tubes were inoculated with 460.5 μL total volume of 45% Bolton broth, 50% bovine plasma, 5% laked horse red blood cells, and 10^6^ CFU of either SA, PA, or both. As PA does not produce coagulase, and thus does not coagulate the media, PAO1 mono-cultures were grown in 15 mL flasks, with 5 mL of media and a scratched pipette tip was used to provide a surface for biofilm development. Cultures were grown for 48 h (96 h for PA alone) at 37°C with shaking at 80 rpm. Following incubation, the established biofilms were transferred to 2 mL homogenizing tubes with 1 mL of 1xPBS and gently rinsed to dislodge any non-attached cells. For the PA mono-cultures, the pipette tips with biofilm coating the outer surface were transferred to new flasks. Subsequently, biofilms were treated with 1 or 7 mL of enzyme solutions (for dual or mono-cultures, respectively) or the vehicle control for 1 h at 37°C with shaking at 80 rpm. Following treatment, the supernatant was removed (this is the “dispersed” fraction). One mL of PBS was added and the remaining biofilms were homogenized at 4 m/s for 60 s in FisherScientific™ 2 mL Pre-Filled Bead Mill Tubes using a FastPrep-24™ MP Biomedical Benchtop Homogenizer. The remaining biofilms on the pipette tips were submerged in PBS and vortexed for 15–20 s. The supernatant and biofilm fractions were serially diluted and spot plated for CFU enumeration. Percent dispersal was calculated as described in the *in vitro* well-plate assay.

### *Ex vivo* Murine Chronic Wound Model

Our murine chronic wound model has been previously described (Brown and Greenhalgh, 1997; Rumbaugh et al., 2009; Wolcott et al., 2010; Dalton et al., 2011; Fleming et al., 2017). Briefly, mice were anesthetized by intraperitoneal injection of sodium pentobarbital. After a surgical plane of anesthesia was reached, the backs were shaved and administered a full thickness, dorsal, 1.5- by 1.5-cm excisional skin wound to the level of panniculus muscle with surgical scissors. Wounds were then covered with a semipermeable polyurethane dressing (Opsite dressing; Smith and Nephew), under which 10^4^ CFU of PA, SA, or 1:1 ratio of PA and SA (in 100 μL PBS) were injected into the wound bed. At 48 h post-infection the mice were euthanized, and the wound beds were harvested for *ex vivo* GH treatments. The harvested wound tissue was cut into 4-sections and each were treated with either 1 mL PBS, amylase, cellulase or amylase and cellulase for 2 h at 37°C, with shaking at 80 rpm. Afterwards, the supernatant containing the dispersed cells was serially diluted and spot plated for CFU enumeration. One mL of PBS was added to the remaining tissue, which was homogenized at 5 m/s for 60 s, then serially diluted and spot plated. Percent dispersal was calculated as described above.

## Results

### The Ability of GHs to Disperse Biofilm Cells Differs in Mono- vs. Poly-Microbial Biofilms

**Figure 1:**
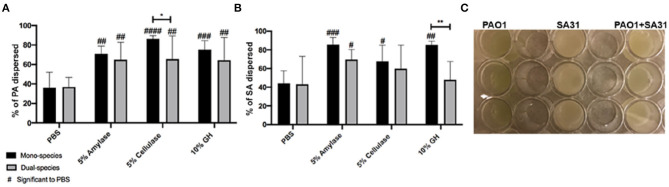
Glycoside hydrolases disperse *P. aeruginosa* and *S. aureus* biofilms. **(A)** *P. aeruginosa* PAO1 mono- and dual-species biofilms, and **(B)** *S. aureus* SA31 mono- and dual-species biofilms. **(C)** Example of *in vitro* well-plate model. Biofilms established in a 24-well cell culture plate for 48 h were treated with PBS (vehicle control), 5% amylase, 5% cellulase, or 10% GH containing both enzymes for 2 h. Percent dispersal was calculated as follows: (CFU in supernatant/ CFU in supernatant + CFU in biofilm). Two-way ANOVA was used to determine significance comparing enzyme to PBS ^#^*P* < 0.05; ^##^*P* < 0.01; ^###^*P* < 0.001; ^####^*P* < 0.0001. Two-way ANOVA was also used to determine significance comparing mono- and dual-species biofilm. *P < 0.05; **P < 0.01; *n* = 5.

In order to determine if the efficacy of GH treatments to disperse PA biofilm varies depending on environment or the presence of SA, we grew PA and SA alone or together in three different biofilm models. The models chosen are standardly used in our laboratory (Brown and Greenhalgh, 1997; Sun et al., 2008; Rumbaugh et al., 2009; Wolcott et al., 2010; Dalton et al., 2011; Deleon et al., 2014; Fleming et al., 2017) and represent a continuum of less to more clinically relevant. We first tested the ability of α-amylase and cellulase, or both combined, to disrupt SA and PA mono- or poly-microbial biofilms grown in a 24-well cell culture plate. After 48 h of bacterial growth, the unattached cells were removed, and the adhered biofilm was rinsed with 1xPBS. The biofilms were then treated with PBS (negative control), 5% α-amylase, 5% cellulase, or 10% GH solution, including a 1:1 ratio of both enzymes, for 2 h. The dispersed cells in the supernatant were removed and the remaining biofilm was sonicated. Both the supernatant and biofilm cells were serially diluted and spot plated on differential media (*Pseudomonas* isolation agar and *Staphylococcus* medium 110) to measure SA and PA CFU and calculate “percent dispersed” for each species. The results of this experiment are shown in Figure 1. The data from this experiment were broken up into panels A and B. Figure 1A compares PA from mono-species biofilms and biofilms where it was grown with SA. The SA data are shown in Figure 1B and compares SA from mono-species biofilms and biofilms where it was grown with PA. Thus, data from the same dual-species biofilms are shown in both panels, but panel A shows only the PA dispersal and panel B shows only the SA dispersal.

Using this simple, well-plate biofilm model we saw that all three treatments significantly dispersed PA in comparison to the PBS treatment, whether or not it was in co-culture with SA (Figure 1A). However, we saw a small, but significant (*p* = 0.0412) reduction in the efficacy of cellulase to disperse PA when SA was present. For SA dispersal, all the treatments were significantly higher in comparison to PBS when SA was alone (Figure 1B). However, the addition of PA resulted in a significant reduction in efficacy of the GH treatment.

We speculate that when PA and/or SA are grown in this model they are not well adhered to the polystyrene surface. This is exemplified in the efficacy of just a PBS treatment to disperse 40% of the cells. It should be noted that we used non-tissue culture treated well-plates for our biofilm assays. Non-treated plates are more hydrophobic than tissue-treated plates. Since a majority of the EPS constituents are hydrophilic, it goes to reason that the biofilms would be more robust in a tissue-treated plate, but we have not compared either biofilm formation or the efficacy of GH treatment between the two. Despite the loose adherence, we did see a significant increase in dispersal after all GH treatments in comparison to PBS, indicating that the enzymes had some effect. These results suggest unexpected enzymatic activity for SA. This activity could be due to promiscuous binding of GHs to various polysaccharide linkages or perhaps the presence of a yet undescribed polysaccharide.

### Cellulase Is Not Effective Against SA in a Wound Microcosm Model

**Figure 2:**
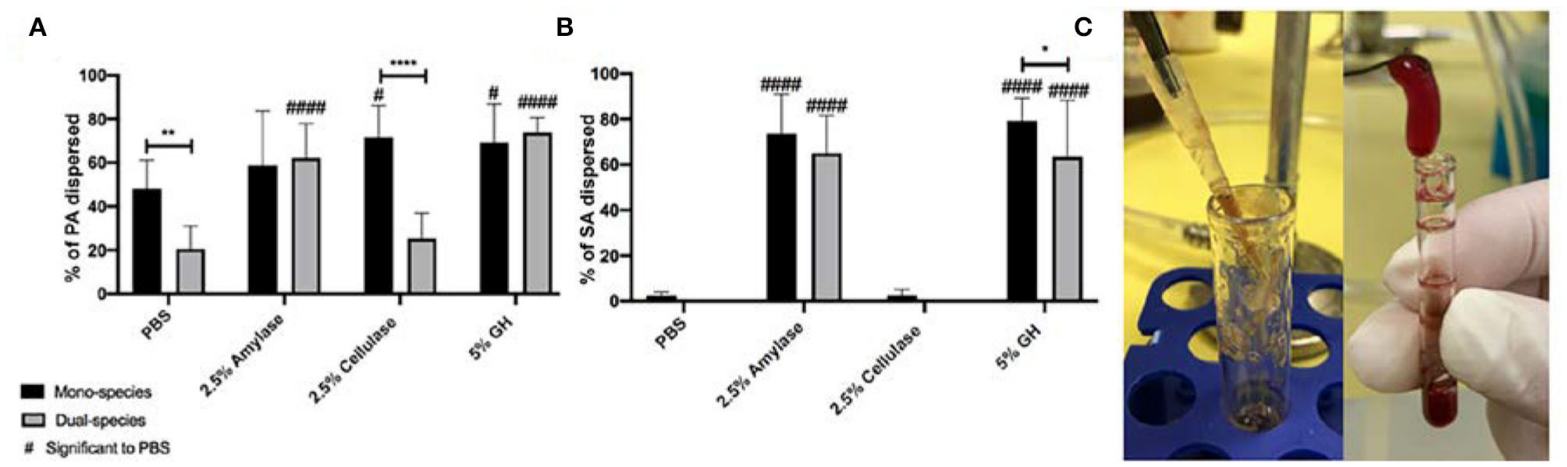
Glycoside hydrolases disperse *P. aeruginosa* and *S. aureus* biofilms in a wound microcosm model. **(A)** *P. aeruginosa* PAO1 mono- and dual-species biofilms, and **(B)** *S. aureus* SA31 mono- and dual-species biofilms. **(C)** Image of PAO1 mono-species wound microcosm model (left) and SA31 in mono- and dual-species microcosm model (right). Biofilms were grown for 48 h then treated with PBS (vehicle control), 2.5% amylase, 2.5% cellulase, or 5% GH containing both enzymes for 1 h. Percent dispersal was calculated as follows: (CFU in supernatant/ CFU in supernatant + CFU in biofilm). Two-way ANOVA was used to determine significance comparing enzyme to PBS ^#^*P* < 0.05; ^####^*P* < 0.0001, or mono- to dual species biofilm *P < 0.05; **P < 0.01; ****P < 0.0001; *n* = 5.

Next, we tested glycoside hydrolases in an *in vitro* microcosm model that mimics a wound-like environment. This model incorporates a “wound-like” media composed of a -chopped-meat-based media, supplemented with heparinized bovine plasma and horse red blood cells. It has been used by us and others to grow poly-microbial biofilms, which reflect the microbial populations of human wound infections (Sun et al., 2008, 2009; Dalton et al., 2011; Trivedi et al., 2017). In this model, the bacterial species of interest are inoculated into the “wound-like” media and incubated aerobically at 37°C. Under static conditions, most of the liquid media coagulates after about 18 h of growth (Figure 2C), due to the ability of coagulase-positive SA to activate the coagulation cascade. SA secretes staphylocoagulase, which binds to prothrombin, forming a complex called staphylothrombin, which then converts soluble fibrinogen to strands of insoluble fibrin (Zajdel et al., 1975). Several SA clumping factors are responsible for binding to fibronectin and fibrinogen (Rivera et al., 2007). Therefore, in this model, we postulate that the bacteria adhere to and reside within host-derived matrix components and this is supported by previous imaging (Deleon et al., 2014).

After 48–96 h of growth, the biofilms were rinsed with PBS and treated for 1 h with either 1x PBS, 2.5% amylase, 2.5% cellulase, or both (5% GH). The supernatant was removed and 1x PBS was added to the remaining biofilm. The biofilms were then homogenized, and both the supernatant and biofilms were serially diluted and spot plated on differential media to enumerate CFU and determine percent dispersal of each species (Figure 2).

In this model we saw that PA alone did not exhibit significant dispersal above PBS for amylase treatment, but cellulase and GH treatments were significantly higher than PBS (Figure 2A). When PA was grown with SA we saw a significant decrease in the ability of PBS to disperse PA. This is interesting and likely indicates that PA is more strongly adhered in general when SA is present. In comparison to this PBS control, a significantly higher number of PA from the dual-species biofilms were dispersed by amylase and GH. Interestingly, when PA was grown with SA in the wound microcosm, it was no longer dispersed by cellulase.

SA biofilms grown in the wound microcosm model were very resistant to dispersal by PBS, likely indicating that they are strongly adhered (Figure 2B). Amylase significantly dispersed SA whether or not it was grown with PA, as did GH, albeit there was a small, but significant reduction (*p* = 0.04) when PA was present. PA was only significantly more susceptible to dispersal with amylase when SA was present. One potential explanation for this result is that SA is the primary biofilm former in this model, thus when SA is dispersed PA is also affected. It's also possible that the expression of PA exopolysaccharides is altered when SA is present in such a way that increases susceptibility to amylase. Strikingly, the ability of cellulase to disperse SA was completely abated in this model. This may be due to a change in the manner by which SA is adhered. In the *in vitro* well-plate model, biofilm adherence will be to the polystyrene surface, or to other cells, presumably facilitated by the production of EPS. As cellulase can disperse cells in this model, we assume that there are susceptible target bonds present. However, in the wound microcosm, the presence of fibrin provides another substrate for adherence. SA possess several fibrin-binding proteins that likely facilitate a tight bond. It is possible that the cellulase-susceptible EPS component produced by SA in the well-plate model, is not produced in the wound microcosm model. As the ability for cellulase to disperse PA was also absent in dual-culture it is possible that PA is able to alter its biofilm forming strategy.

### The Microcosm Model Is More Representative of GH Efficacy *in vivo*

**Figure 3:**
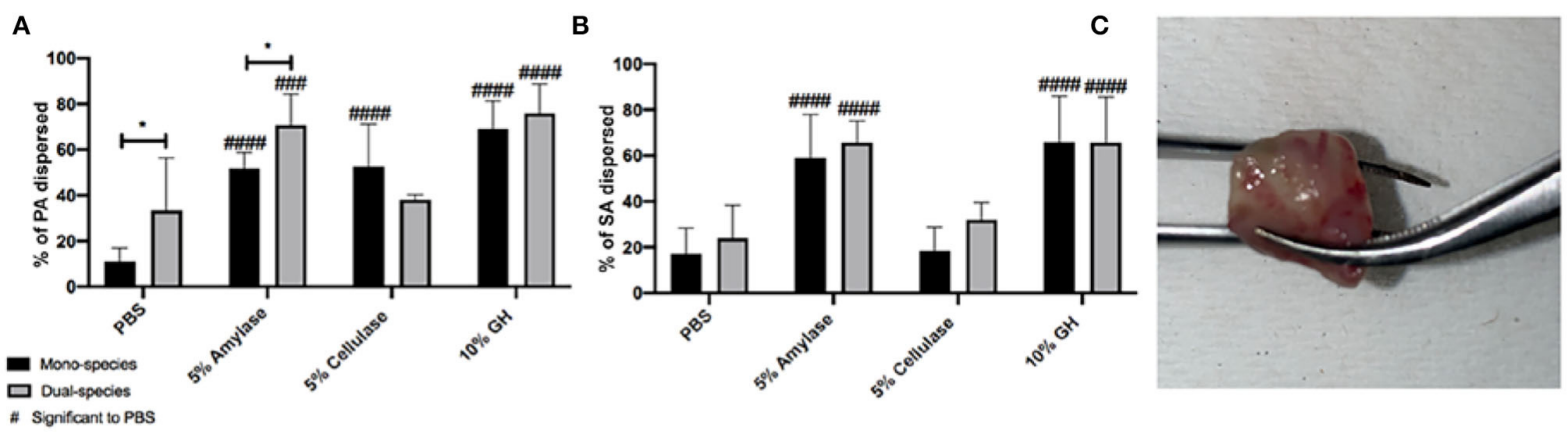
Glycoside hydrolases disperse *P. aeruginosa* and *S. aureus* biofilms from infected wound tissue. **(A)** *P. aeruginosa* PAO1 mono- and dual-species biofilms, and **(B)** *S. aureus* SA31 mono- and dual-species biofilms. **(C)** Image of infected tissue collected from murine wound bed. Infected wound tissue was treated with PBS (vehicle control), 5% amylase, 5% cellulase, or 10% GH containing both enzymes for 2 h. Percent dispersed was calculated as follows: (CFU in supernatant/ CFU in supernatant+ CFU in biofilm). Two-way ANOVA was used to determine significance comparing enzyme to PBS ^###^*P* < 0.001; ^####^*P* < 0.0001, or mono- to dual-species biofilm *P < 0.05; *n* = 5.

Lastly, we tested the efficacy of glycoside hydrolases to degrade biofilms grown *in vivo*. For these experiments we used a murine chronic wound model as previously described (Brown and Greenhalgh, 1997; Rumbaugh et al., 2009; Wolcott et al., 2010; Dalton et al., 2011; Fleming et al., 2017). Wounds were infected with PA, SA, or a 1:1 ratio of both. After 48 h of infection, mice were euthanized, and the infected wound tissue was collected for *ex vitro* treatments. Excised wound tissue was divided and treated with PBS, 5% α-amylase, 5% cellulase, or a combination of both for 2 h. After the allotted treatment time, the supernatant was removed and the remaining biofilms were homogenized. Both the supernatant and biofilms were serially diluted and spot plated to enumerate CFU followed by percent dispersal calculations (Figure 3).

In this model, we saw a significant difference in the ability of PBS to disperse PA in mono vs. dual-species infections, possibly indicating that PA makes more robust biofilms when it is alone, vs. when SA is present. All three enzyme treatments significantly dispersed PA from mono-infections; however, similar to what we saw with the wound microcosm model, cellulase lost its ability to disperse PA when SA was also present in the infection (Figure 3A). Cellulase was also unable to disperse SA from wound-associated biofilms whether it was alone or with PA (Figure 3B), and this was also similar to results obtained with the microcosm model.

**Figure 4:**
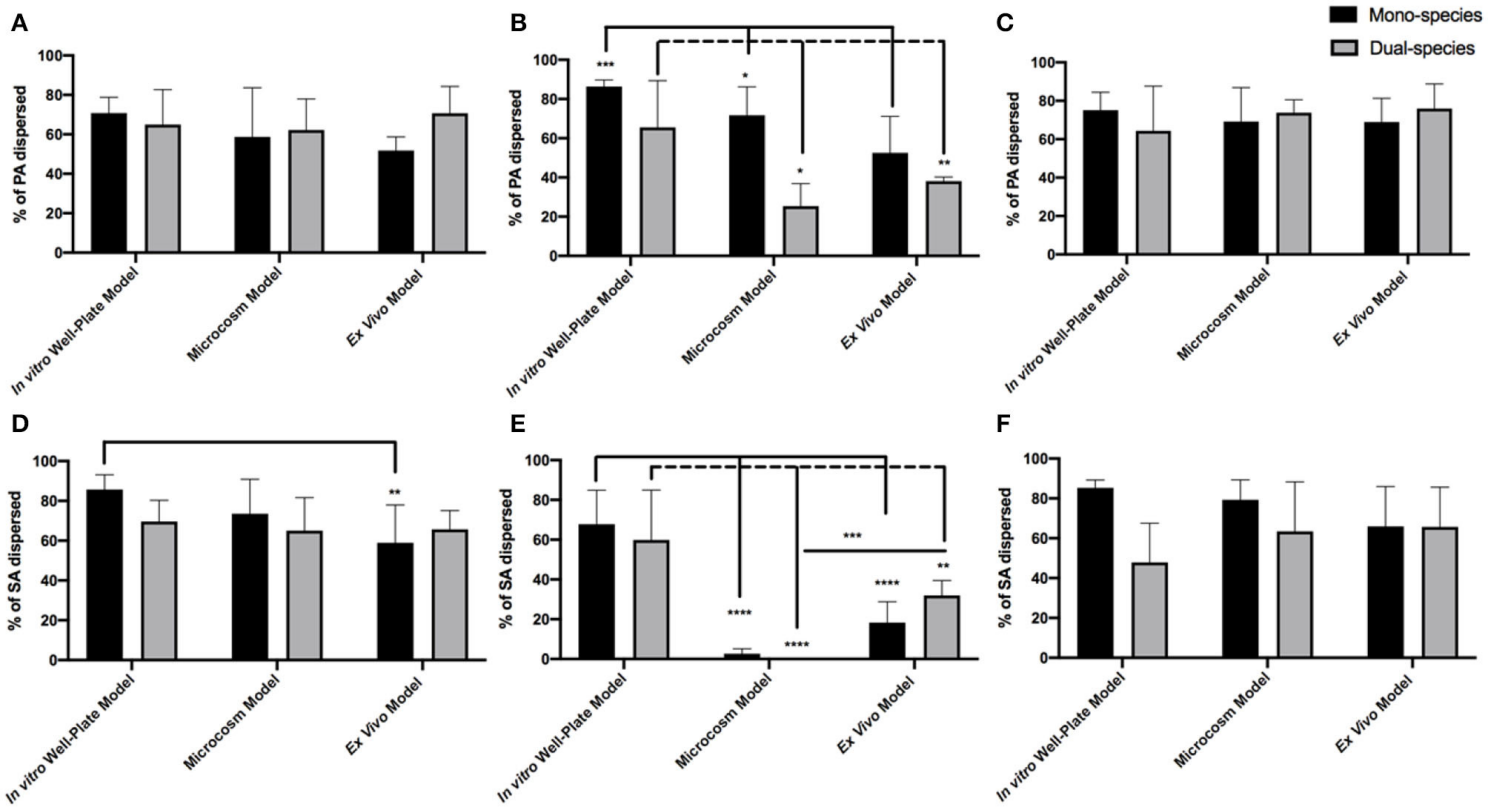
GH percent dispersal by model utilized in PAO1 **(A–C)** and SA31 **(D–F)** mono- and dual-species biofilms. Dispersal was compared by treatment with either Amylase **(A,D)**, Cellulase **(B,E)**, or both **(C,F)**. Percent dispersed was calculated as follows: (CFU in supernatant/ CFU in supernatant+ CFU in biofilm). Two-way ANOVA was used to determine significance comparing results between models. *P < 0.05; **P < 0.01; ***P < 0.001; ****P < 0.0001; *n* = 5.

In order to better understand how the efficacy of our different enzyme treatments change based on the environment in which PA, SA, or both are grown, we compared enzyme activity between models (Figure 4). For amylase there was little difference in efficacy between models. We saw significant dispersal of both PA and SA, whether they were alone or together in all models tested (Figure 4A). We did observe a small, but significant, decrease in the dispersal of SA from mono-infections (*ex vivo*) in comparison to the *in vitro* well-plate model, but overall the results were consistent between models. Based on our data, amylase alone is a good disperser of both SA and PA even if they are in a co-infection. However, the story is quite different for cellulase. Cellulase appears to disperse relatively well for both PA and SA in well-plates, but almost completely loses activity against SA in more clinically relevant models that incorporate host factors (Figure 4E). Interestingly, this also appears to be true for PA, but only when it is with SA (Figure 4B). The efficacy of the GH formulation, which combines amylase and cellulase, is also relatively consistent between models, suggesting that the amylase portion is likely responsible for most of the activity.

One possible explanation for this result is that the activity of cellulase is inhibited by proteolytic blood components in the microcosm model and *ex vivo* tissue. However, as shown in Figure 4B, cellulase treatment of PAO1 alone in the microcosm alone was still effective, suggesting the enzyme is still active. Another possibility is that the EPS components of the biofilm change when both PA and SA are together in such a way that cellulase becomes ineffective or an EPS structural change induces steric hindrance and inhibits cellulase from interacting with the linkage target.

## Discussion

It has been well documented in the literature that the growth environment affects biofilm formation as well as the susceptibility of bacteria to many antimicrobials (Stanley and Lazazzera, 2004; Karatan and Watnick, 2009; Mah, 2012). Yet, we still have little understanding of these effects. For example, we do not have a good understanding of how biofilm formation or EPS composition varies when bacteria are placed in different environments or when other microbes are present. As so many phenotypic traits are influenced by environmental factors, it is fair to speculate that the composition of biofilm EPS will also vary.

Here we tested whether the efficacy of GHs differed depending on the growth environment. Our data suggest that in the *in vitro* well-plate model, both SA and PA are loosely adhered to the polystyrene surface. Therefore, the enzymes (and the PBS control) easily break-up the biofilm and induce a high level of dispersal. However, in the microcosm and *ex vivo* models, SA in particular displays a tighter attachment, likely due to other factors than just EPS, such as the activity of binding proteins. It also appears that, in the microcosm model, PA may benefit from the tight adherence of SA, as PA is more resistant to dispersal as well.

Interestingly, cellulase treatment significantly dispersed SA in the *in vitro* well-plate model, but not in the microcosm or *ex vivo* models (Figure 4E). Furthermore, cellulase did not disperse PA as well when it was with SA in these latter models. It is possible that EPS production by SA, PA or both, changes depending on the model, making it less susceptible to cellulase. For example, in the microcosm model, PA may produce an EPS dominated by Pel, which does not possess the linkages targeted by cellulase, but when grown in the well-plate model, more alginate is produced. We also saw that amylase significantly dispersed SA whether it was alone or with PA in all models. While PNAG does not possess bonds targeted by amylase, perhaps SA produces another exopolysaccharide(s) with α- 1,4 linkages that has yet to be described. It is additionally possible that amylase could promiscuously bind linkages other than α- 1,4. To further investigate whether EPS composition changes depending on environment, the expression of genes involved in the production of PA and SA polysaccharides in each model could be measured. Another option would be to use PA and SA strains with mutations in key polysaccharide genes and determine whether the deletion of specific genes affects dispersal susceptibility. Lastly, staining or immunohistochemistry of mono- and dual-species biofilms grown in different models could reveal EPS alterations, but these experiments are challenging because there are few reagents available that are specific for bacterial polysaccharides.

When thinking about testing the efficacy of therapeutic agents, it is important to perform experiments in models that recapitulate the infection environment as closely as possible. Ideally, efficacy tests are performed *in vivo*, but using *in vitro* models for early stage optimization of therapeutics is not only economical, but humane. In this study we examined the efficacy of one experimental therapeutic agent, GH, in an *in vitro* model that attempts to recapitulate the wound environment. Overall, the dispersal results obtained in the wound microcosm more closely resembled those obtained in *ex vivo*-infected tissue, providing a good predicative model for GH activity. Our data also suggest that the nature of the EPS produced by PA and SA differs depending on the model, which leaves the door open for future research and perhaps provides another utility for GHs.

## Data Availability Statement

The raw data supporting the conclusions of this article will be made available by the authors, without undue reservation.

## Ethics Statement

The animal study was reviewed and approved by the Institutional Animal Care and Use Committee of Texas Tech University Health Sciences Center (protocol number 07044). This study was carried out in strict accordance with the recommendations in the Guide for the Care and Use of Laboratory Animals of the National Institutes of Health.

## Author Contributions

All authors contributed to designing the experiments and writing the manuscript. WR and GW executed the experiments.

## Conflict of Interest

The authors declare that the research was conducted in the absence of any commercial or financial relationships that could be construed as a potential conflict of interest.
